# Efficacy of Surgery in Patients with Stage I Primary Parotid Gland Lymphoma: A Population-Based Study

**DOI:** 10.1155/2022/4977600

**Published:** 2022-11-28

**Authors:** Xiaoyu He, Dongxu Gang, Xiaoyuan Zhang, Hanyi Ding, Songfu Jiang

**Affiliations:** Department of Hematology, The First Affiliated Hospital of Wenzhou Medical University, Wenzhou, China

## Abstract

**Background:**

Limited and controversial evidence is available on the efficacy of surgery for patients with stage I primary parotid gland lymphoma. Thus, we aimed to investigate whether surgery can enhance the prognosis of patients with stage I primary parotid gland lymphoma using large sample data.

**Methods:**

From 1998 to 2015, we searched the Surveillance, Epidemiology, and End Results (SEER) program database and extracted information regarding patients with stage I primary parotid gland lymphoma; we classified these patients into surgery and nonsurgery groups. We calculated overall survival (OS) and cancer-specific survival (CSS) using Kaplan–Meier curves and log-rank testing. Propensity score matching (PSM) analysis was also used to further account for confounding variables before comparing the OS and CSS again. We used the COX proportional hazard regression model in both multivariate and univariate analyses.

**Results:**

We enrolled 918 patients with stage I primary parotid gland lymphoma, among which 656 (71.5%) patients underwent surgery. Before PSM, the surgery group had better OS (hazard ratio (HR) = 0.673, 95% confidence interval (CI): 0.519–0.873, and *p*=0.003) and CSS (HR = 0.595, 95% CI: 0.403–0.879, and *p*=0.008) than the nonsurgery group. After PSM, surgery was still a beneficial factor for OS (HR = 0.569, 95% CI: 0.399–0.810, and *p*=0.002) and CSS (HR = 0.384, 95% CI: 0.220–0.669, and *p*=0.001). Furthermore, in univariate and multivariate analyses, total parotidectomy significantly increased OS (*p*=0.001 and *p*=0.021, respectively) and CSS (*p*=0.001 and *p*=0.037, respectively).

**Conclusions:**

In summary, the prognosis of patients with stage I primary parotid gland lymphoma can be significantly improved by surgery. Moreover, total parotidectomy was a protective factor for OS and CSS before and after PSM analysis, suggesting that surgery acts as a significant component in multimodal therapy for early primary parotid gland lymphoma.

## 1. Introduction

Primary parotid gland lymphoma is a rare type of head and neck lymphoma, with a total incidence of roughly 0.3% of all cancer cases, 2%–5% of salivary gland tumors, and 5% of extranodal lymphomas [1–3]. Patients with primary parotid gland lymphoma often have a lower-grade lymphoma and better prognosis than those with other extranodal lymphomas [4, 5]. Primary parotid gland lymphoma is described by its asymptomatic progress in the parotid gland for 4–6 months [6]. Swelling of both parotid glands, cervical lymphadenopathy, pain, and facial nerve paralysis are the other symptoms [7, 8]. The clinical outcome of parotid lymphoma appears similar to other benign parotid swellings, thus making the diagnosis difficult. Lymphomas are commonly overlooked in a new parotid mass in preoperative evaluations [9]. The presence of autoimmune conditions, such as Sjögren syndrome, is linked to an elevated risk of lymphoma (44%). Moreover, these conditions increase the risk of lymphomas of the parotid gland, particularly marginal zone lymphoma [10].

Radiotherapy, chemotherapy, or both may be used to modify treatment to the specific grade and histologic type; this provides a positive prognosis in most cases [11–13]. However, there have been controversies over the role of surgery in primary parotid gland lymphoma. Surgical excision can enhance OS [14–16]; however, some studies found that it did not affect patients' survival [6]. Furthermore, surgery acts only as a diagnostic tool [17–19].

We used population-based data from the Surveillance, Epidemiology, and End Results (SEER) database to determine whether surgery for stage I primary parotid gland lymphoma was effective. We also assessed the survival effects of various surgical methods.

## 2. Methods

### 2.1. Data Sources

We used the SEER program database of the National Cancer Institute, which gathers data on cancer incidence and survival from 15 states and covers roughly 34.6% of the US population. Using SEER^*∗*^stat software package 8.3.5, we extracted eligible cases from the SEER-18 registry (with modified treatment fields). The data were free to download from the SEER database and thus did not require informed consent from patients; we filed a request to the SEER database project and obtained approval. From 1998 to 2015, we gathered the following information from the SEER database for all patients with primary parotid gland lymphoma: demographics, pathological types, Ann Arbor stage I, treatment information (surgery, radiation, and chemotherapy), and survival. The following patients were excluded: (1) those who did not have primary parotid gland lymphoma as the sole malignancy, (2) those who did not provide information on race or marital status, (3) those who did not have a clear surgical therapy, and (4) those who had a survival time of zero.

### 2.2. Statistical Analysis

Descriptive statistics were used for the assessment of the demographic and tumor features of the patients. Categorical variables were assessed using the chi-square test. The Cox proportional risk model was used to assess characteristics that were independent predictors of OS and CSS. The multivariate Cox analysis included factors that were substantially linked with prognosis in the univariate Cox analysis. Moreover, the hazard ratios (HRs) and 95% confidence intervals (CIs) were computed using the Cox proportional hazards models. A Kaplan–Meier survival curve was created using this technique; the log-rank test was used to assess the difference between the survival curves.

An analysis of propensity scores on a one-to-one basis was performed using R statistical language to reduce baseline differences between the two groups. All statistical analyses were performed using R statistical language (Version R 4.2.1). *p* < 0.05 was considered statistically significant when using two-sided tests.

## 3. Results

### 3.1. Comparing Baseline Data

We included 918 patients with stage I primary parotid gland lymphoma, of which 656 (71.5%) underwent surgery. The proportion of patients aged ≤65 years (74.5%, *n* = 366), that of females (70.6%, *n* = 398), that of Caucasians (71.6%, *n* = 554), and that of married patients (74.1%, *n* = 401) was higher. There was an increase in the proportion of patients who were diagnosed over time. Specifically, 29.4% (*n* = 270) of patients were diagnosed in 1998–2003, whereas 35.5% (*n* = 326) of patients were diagnosed in 2010–2015. In total, 97.9% (*n* = 899) patients had unilateral tumors, and only 2.1% (*n* = 19) had bilateral tumors. The most common histology was mucosal-associated lymphoid tissue (MALT), with 46.5% of cases. The minority of patients underwent radiotherapy (47.5%, *n* = 436) and chemotherapy (30.6%, *n* = 281) (Table 1).

### 3.2. Matching Based on Propensity Scores

All patient variables were adjusted using 1 : 1 PSM analysis to reduce selection bias. After PSM, the following characteristics of the patients did not differ significantly between the two groups: age (*p*=0.094), sex (*p*=0.631), race (*p*=0.261), marital status (*p*=0.403), year of diagnosis (*p*=0.412), laterality (*p*=0.249), radiotherapy (*p*=1.000), and chemotherapy (*p*=0.921) (Table 2).

### 3.3. OS and CSS Comparison

**Figure 1:**
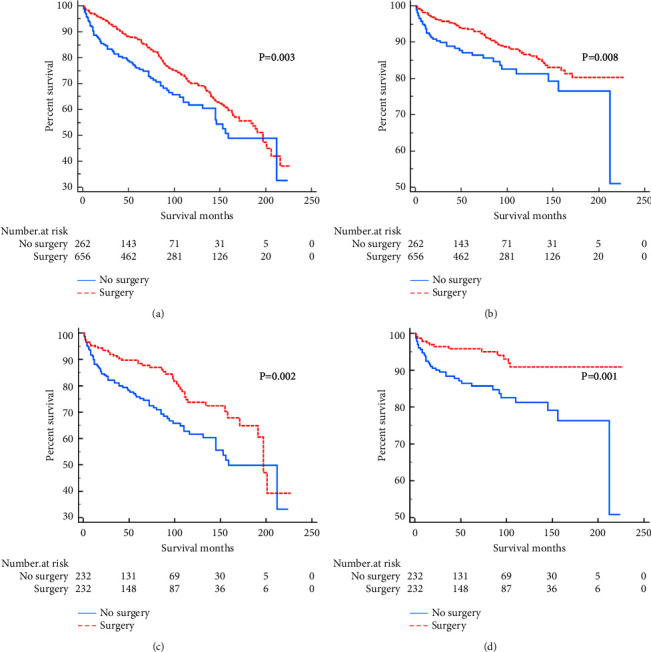
Survival curve for OS and CSS comparison. OS (a) and CSS (b) for patients with primary parotid gland lymphoma before PSM; OS (c) and CSS (d) for patients with stage I primary parotid gland lymphoma after PSM.

A comparison of OS and CSS of patients with stage I primary parotid gland lymphoma is shown in Table 3. Before PSM, the surgery group had a better OS (HR = 0.673, 95% CI: 0.519–0.873, *p*=0.003) (Figure 1(a)) and CSS (HR = 0.595, 95% CI: 0.403–0.879, *p*=0.008) (Figure 1(b)) than the nonsurgery group. After PSM, the OS (HR = 0.569, 95% CI: 0.399–0.810, *p*=0.002) (Figure 1(c)) and CSS (HR = 0.384, 95% CI: 0.220–0.669, *p*=0.001) (Figure 1(d)) of the two groups of patients still differed significantly.

### 3.4. OS and CSS Assessment Using Univariate and Multivariate Analyses

Table 4 shows the findings of a survival study of individuals with stage I primary parotid gland lymphoma. Univariate analysis revealed that an age of >65 years (HR = 7.335, 95% CI: 5.397–9.969, *p* < 0.001), diffuse large B-cell lymphoma (DLBCL) (HR = 2.548, 95% CI: 1.852–3.505, *p* < 0.001), follicular lymphoma (FL) (HR = 1.572, 95% CI: 1.141–2.165, *p*=0.006), and other lymphomas (HR = 1.605, 95% CI: 1.133–2.275, *p*=0.008) were the risk factors for the OS of patients with stage I primary parotid gland lymphoma. Conversely, being black (HR = 0.571, 95% CI: 0.339–0.962, *p*=0.035), of another race (HR = 0.481, 95% CI: 0.262–0.881, *p*=0.018), and married (HR = 0.611, 95% CI: 0.480–0.776, *p* < 0.001); year of diagnosis being within 2010–2015 (HR = 0.531, 95% CI: 0.345–0.817, *p*=0.004); and undergoing superficial parotidectomy (HR = 0.687, 95% CI: 0.490–0.966, *p*=0.031) and total parotidectomy (HR = 0.598, 95% CI: 0.437–0.818, *p*=0.001) were protective factors for the OS of patients with stage I primary parotid gland lymphoma. However, radiation, chemotherapy, sex, laterality, and local tumor excision did not have a statistically significant effect (*p* > 0.05).

When different statistically significant variables were incorporated into a regression model for multivariate analysis, the results revealed that an age of >65 years (HR = 7.248, 95% CI: 5.287–9.936, *p* < 0.001), DLBCL (HR = 1.900, 95% CI: 1.370–2.636, *p* < 0.001), and other lymphomas (HR = 1.657, 95% CI: 1.157–2.373, *p*=0.006) were risk factors for the OS of patients with stage I primary parotid gland lymphoma. Conversely, being of another race (HR = 0.469, 95% CI: 0.255–0.864, *p*=0.015) and married (HR = 0.646, 95% CI: 0.505–0.828, *p*=0.001); year of diagnosis being within 2010–2015 (HR = 0.536, 95% CI: 0.347–0.829, *p*=0.005); and undergoing superficial parotidectomy (HR = 0.697, 95% CI: 0.492–0.989, *p*=0.043) and total parotidectomy (HR = 0.682, 95% CI: 0.492–0.945, *p*=0.021) were protective factors for the OS of patients with stage I primary parotid gland lymphoma. Being black (HR = 0.725, 95% CI: 95% CI: 0.425–1.237, *p*=0.239) or having FL (HR = 1.189, 95% CI: 0.860–1.644, *p*=0.295) were not statistically significant.

The findings of the CSS survival analysis of patients with stage I primary parotid gland lymphoma are shown in Table 5. According to univariate analysis, an age of >65 years (HR = 3.475, 95% CI: 2.323–5.198, *p* < 0.001) and having DLBCL (HR = 4.045, 95% CI: 2.413–6.783, *p* < 0.001), FL (HR = 1.973, 95% CI: 1.131–3.442, *p*=0.017), and other lymphomas (HR = 3.630, 95% CI: 2.166–6.083, *p* < 0.001) were risk factors for the CSS of patients. Conversely, being married (HR = 0.549, 95% CI: 0.378–0.795, *p*=0.002), year of diagnosis being within 2010–2015 (HR = 0.410, 95% CI: 0.218–0.772, *p*=0.006), and undergoing total parotidectomy (HR = 0.429, 95% CI: 0.258–0.715, *p*=0.001) and radiation (HR = 0.663, 95% CI: 0.454–0.969, *p*=0.034) were protective factors for the CSS of patients. Sex, race, laterality, chemotherapy, local tumor excision, and superficial parotidectomy were not statistically significant (*p* > 0.05).

Multivariate analysis was conducted similarly, and the results indicated that an age of >65 years (HR = 3.634, 95% CI: 2.389–5.528, *p* < 0.001) and having DLBCL (HR = 3.471, 95% CI: 2.036–5.917, *p* < 0.001) and other lymphomas (HR = 3.513, 95% CI: 2.061–5.989, *p* < 0.001) were the risk factors for the CSS of patients. Conversely, being married (HR = 0.663, 95% CI: 0.453–0.971, *p*=0.035), year of diagnosis being within 2010–2015 (HR = 0.397, 95% CI: 0.209–0.753, *p* < 0.005), and undergoing total parotidectomy (HR = 0.573, 95% CI: 0.339–0.968, *p*=0.037) and radiation (HR = 0.607, 95% CI: 0.410–0.898, *p*=0.012) were protective factors for the CSS of patients. Having FL (HR = 1.665, 95% CI: 0.951–2.916, *p*=0.074) did not have any statistically significant difference.

### 3.5. Impact of Surgical Techniques on OS and CSS

**Figure 2:**
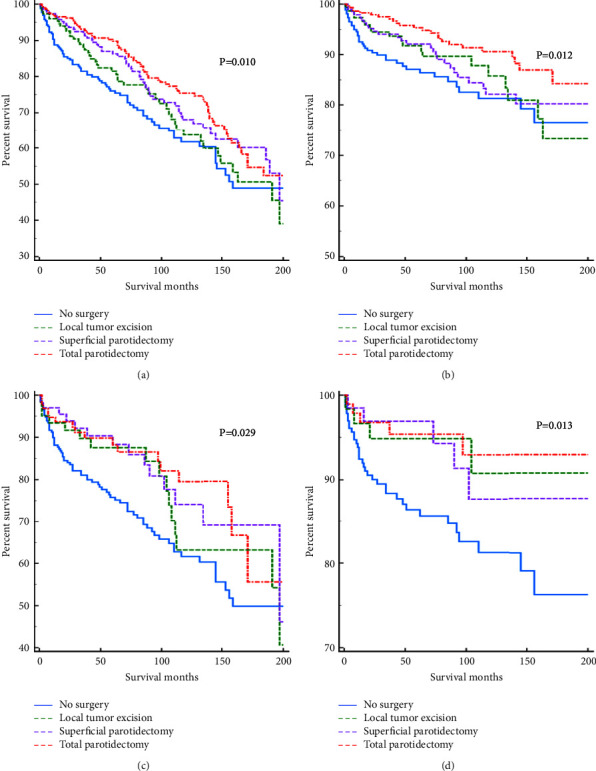
The effect of different surgical procedures on OS and CSS. OS (a) and CSS (b) for patients with primary parotid gland lymphoma before PSM; OS (c) and CSS (d) for patients with stage I primary parotid gland lymphoma after PSM.

**Figure 3:**
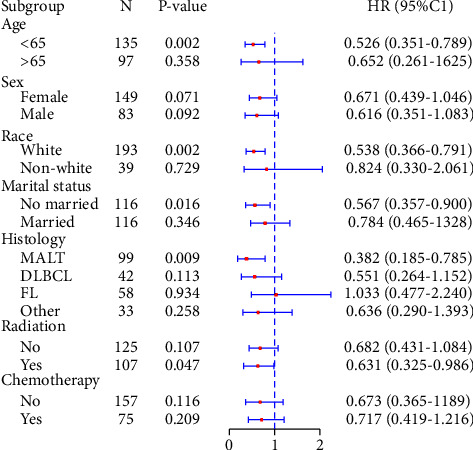
Forest plot of the predictors of prognosis in patients who underwent surgery.

As shown in Figure 2, surgery can significantly improve prognosis. Specifically, the effects of different surgical procedures on OS and CSS are shown in Table 6. In patients with stage I primary parotid gland lymphoma, total parotidectomy improved OS (HR = 0.592, 95% CI: 0.432–0.809, *p*=0.001) and CSS (HR = 0.426, 95% CI: 0.256–0.709, *p*=0.001) compared with the nonsurgery group; superficial parotidectomy improved OS (HR = 0.673, 95% CI: 0.519–0.873, *p*=0.003) but did not significantly differ in CSS (*p*=0.207). Local tumor excision did not show any significant difference in both groups for both OS and CSS (*p*=0.257 and *p*=0.255, respectively). After PSM, total parotidectomy had superior OS (HR = 0.533, 95% CI: 0.319–0.892, *p*=0.017) and CSS (HR = 0.319, 95% CI: 0.125–0.815, *p*=0.017) compared with no surgery; conversely, superficial parotidectomy only had superior OS (HR = 0.563, 95% CI: 0.318–0.996, *p*=0.048). There was no significant difference in OS and CSS between the local tumor resection group and the nonsurgery group (*p*=0.173 and *p*=0.065, respectively). Furthermore, sex and chemotherapy had little effect on the results of individuals treated with surgery (Figure 3).

## 4. Discussion

The early diagnosis of primary parotid gland lymphoma is still challenging as the condition can be easily misdiagnosed or even left unnoticed because the incidence of primary parotid gland lymphoma is extremely low and the initial clinical symptoms are untypical. The main diagnostic tool for primary parotid gland lymphoma is surgery since the accuracy of fine-needle aspiration biopsy is low and the radiological features are not obvious [20, 21]. Magnetic resonance imaging, computed tomography, and ultrasound do not provide much additional information at the onset of parotid swelling [22]. Parotidectomy is highly recommended both for treating the tumor and for histologically diagnosing the tumor for further follow-up planning [2].

Depending on its grade and stage, the treatment of primary parotid gland lymphoma is determined. The majority of parotid lymphomas are low-grade and locally confined Ann Arbor lymphomas of Stage I or II that can be treated with surgery, adjuvant radiation or chemotherapy, or both, depending on their stage and grade [14, 23–26]. Surgery is performed in most cases where systemic treatment is required; however, the procedure is categorized as a diagnostic measure [1, 9, 27–30]. Conversely, surgical excision effectively reduces the tumor size and improves the prognosis. The five-year survival rate reached 61% for parotid lesions with a diameter of >6 cm and increased to 87% when the largest diameter was <6 cm [31, 32]. Furthermore, total excision of all known lymphomas, such as stomach lymphoma, may be curative for some subtypes of lymphomas [33, 34]. The treatment method did not affect the OS of patients with low-grade primary parotid gland lymphoma. In most cases, parotidectomy alone might be considered a curative option [24, 25].

The findings of this study indicate that surgical treatment of stage I primary parotid gland lymphoma may provide some survival benefits. The multivariate analysis results revealed that surgery was an independent predictor of living a longer life, in agreement with a previous retrospective analysis, wherein Feinstein et al. analyzed data collected from the SEER database concerning 2,140 patients with parotid lymphoma. They found that patients who underwent surgery had a 35% lower death rate than patients who did not [14]. Vazquez et al. found that there was no statistically significant difference in survival among groups treated with surgery, radiation therapy, or both for MALT lymphoma of the salivary glands [25]. Olivier et al. studied 35 patients diagnosed with Ann Arbor Stage I and II NHL of the parotid gland and reported 90% OS and 71% disease-specific survival at 5 years and 10 years after surgery, with no significant difference between the radiation and surgery groups [35].

Furthermore, we analyzed the effect of different surgical procedures on the outcome. Compared with patients who did not undergo surgery, patients who underwent total parotidectomy had better OS and CSS, but patients with superficial parotidectomy only had better OS. As opposed to previous studies, Mehle et al. found that surgery does not affect the prognosis regardless of whether it is superficial or total parotidectomy [6]. This was probably due to their sample size being small (only 16 subjects), so they could not accurately assess the effects. In the case of malignant tumors without facial nerve involvement that can be completely resected during surgery, superficial parotidectomy was proven to be an effective therapeutic method for unifocal, early-stage parotid gland lymphoma [36]. When the facial nerve is confirmed to be infiltrated preoperatively or during surgery, total parotidectomy combined with resection of the facial nerve is recommended [37]. In the surgery group, the prognosis was better if the patient was younger than 65 years; therefore, age should be considered an important factor in clinical practice when considering whether surgery should be performed. Surgery is recommended for white people, unmarried individuals, and people receiving radiotherapy since they have a better prognosis. Patients diagnosed with parotid lymphoma due to MALT had better prognosis in the surgery group, consistent with previous studies. Early-stage parotid gland MALT lymphomas have an idle behavior and tend to remain localized for long periods, requiring less aggressive treatment [7].

There are several limitations to our study. First, because this was a retrospective study, some selection bias was inevitable; it is possible that patients in the surgery group were healthier and had lower tumor loads than those in the nonsurgery group. Second, there is no detailed information in the SEER database regarding the purpose, timing, and outcome of surgery; the recurrence rates following surgery also remain unclear. Therefore, a multidisciplinary comprehensive evaluation is essential when determining which patients should be recommended for surgery. Nevertheless, the strengths of the study are that this is the first population-based study to assess the efficacy of surgical therapy in patients with early-stage primary parotid gland lymphoma. Furthermore, we drew the study population from a nationally representative dataset, which may have reduced potential selection bias to an extent. Considering that both multivariable and PSM analyses were conducted and that no significant changes were noted in OS, we can affirm that our findings are valid and stable.

## 5. Conclusions

In summary, surgery can considerably improve the OS and CSS of patients with stage I primary parotid gland lymphoma, thus supporting an increased role of surgery in multimodal treatment.

## Figures and Tables

**Table 1:** Comparison of baseline data.

Characteristics	Level	Overall	No surgery	Surgery	*p*
		*N* = 918	*N* = 262 (28.5)	*N* = 656 (71.5)	
Age (%)	≤65	491 (53.5)	125 (25.5)	366 (74.5)	0.032
	>65	427 (46.5)	137 (32.1)	290 (67.9)	

Sex (%)	Female	564 (61.4)	166 (29.4)	398 (70.6)	0.496
	Male	354 (38.6)	96 (27.1)	258 (72.9)	

Race (%)	White	774 (84.3)	220 (28.4)	554 (71.6)	0.545
	Black	82 (8.9)	21 (25.6)	61 (74.4)	
	Other	62 (6.8)	21 (33.9)	41 (66.1)	

Marital status (%)	No married	377 (41.1)	122 (32.4)	255 (67.6)	0.039
	Married	541 (58.9)	140 (25.9)	401 (74.1)	

Year of diagnosis (%)	1998–2003	270 (29.4)	55 (20.4)	215 (79.6)	<0.001
	2004–2009	322 (35.1)	91 (28.3)	231 (71.7)	
	2010–2015	326 (35.5)	116 (35.6)	210 (64.4)	

Histology (%)	MALT	427 (46.5)	97 (22.7)	330 (77.3)	<0.001
	DLBCL	156 (17.0)	66 (42.3)	90 (57.7)	
	FL	194 (21.1)	43 (22.2)	151 (77.8)	
	Other	141 (15.4)	56 (39.7)	85 (60.3)	

Laterality (%)	Right	443 (48.3)	116 (26.2)	327 (73.8)	0.030
	Left	456 (49.7)	136 (29.8)	320 (70.2)	
	Bilateral	19 (2.1)	10 (52.6)	9 (47.4)	

Radiation (%)	No	482 (52.5)	144 (29.9)	338 (70.1)	0.385
	Yes	436 (47.5)	118 (27.1)	318 (72.9)	

Chemotherapy (%)	No	637 (69.4)	162 (25.4)	475 (74.6)	0.002
	Yes	281 (30.6)	100 (35.6)	181 (64.4)	

MALT: mucosal-associated lymphoid tissue, DLBCL: diffuse large B-cell lymphoma, and FL: follicular lymphoma.

**Table 2:** Comparison of baseline data after PSM.

Characteristics	Level	Overall	No surgery	Surgery	*p*
		464	232	232	
Age (%)	≤65	251 (54.1)	116 (50.0)	135 (58.2)	0.094
	>65	213 (45.9)	116 (50.0)	97 (41.8)	

Sex (%)	Female	292 (62.9)	143 (61.6)	149 (64.2)	0.631
	Male	172 (37.1)	89 (38.4)	83 (35.8)	

Race (%)	White	386 (83.2)	193 (83.2)	193 (83.2)	0.261
	Black	49 (10.6)	21 (9.1)	28 (12.1)	
	Other	29 (6.2)	18 (7.8)	11 (4.7)	

Marital status (%)	No married	222 (47.8)	106 (45.7)	116 (50.0)	0.403
	Married	242 (52.2)	126 (54.3)	116 (50.0)	

Year of diagnosis (%)	1998–2003	107 (23.1)	54 (23.3)	53 (22.8)	0.412
	2004–2009	156 (33.6)	84 (36.2)	72 (31.0)	
	2010–2015	201 (43.3)	94 (40.5)	107 (46.1)	

Histology (%)	MALT	195 (42.0)	96 (41.4)	99 (42.7)	0.046
	DLBCL	97 (20.9)	55 (23.7)	42 (18.1)	
	FL	95 (20.5)	37 (15.9)	58 (25.0)	
	Other	77 (16.6)	44 (19.0)	33 (14.2)	

Laterality (%)	Right	213 (45.9)	107 (46.1)	106 (45.7)	0.249
	Left	237 (51.1)	115 (49.6)	122 (52.6)	
	Bilateral	14 (3.0)	10 (4.3)	4 (1.7)	

Radiation (%)	No	251 (54.1)	126 (54.3)	125 (53.9)	1.000
	Yes	213 (45.9)	106 (45.7)	107 (46.1)	

Chemotherapy (%)	No	312 (67.2)	155 (66.8)	157 (67.7)	0.921
	Yes	152 (32.8)	77 (33.2)	75 (32.3)	

CI: confident interval and HR: hazard ratio.

**Table 3:** Comparison of overall survival and cancer-specific survival.

	Characteristics	Overall survival HR (95% CI)	*p*	Cancer-specific survival HR (95% CI)	*p*
Before PSM	No surgery	1		1	
	Surgery	0.673 (0.519, 0.873)	**0.003**	0.595 (0.403, 0.879)	**0.008**

After PSM	No surgery	1		1	
	Surgery	0.569 (0.399, 0.810)	**0.002**	0.384 (0.220, 0.669)	**0.001**

**Table 4:** Univariate and multivariate analysis of overall survival.

Characteristics	Univariate analysis HR (95% CI)	*p*	Multivariate analysis HR (95% CI)	*p*
Age				
≤65	1		1	
>65	7.335 (5.397–9.969)	**<0.001**	7.248 (5.287–9.936)	**<0.001**
Sex				
Female	1			
Male	1.045 (0.817–1.336)	0.726	NA	NA
Race				
White	1		1	
Black	0.571 (0.339–0.962)	**0.035**	0.725 (0.425–1.237)	0.239
Other	0.481 (0.262–0.881)	**0.018**	0.469 (0.255–0.864)	**0.015**
Marital status				
No married	1		1	
Married	0.611 (0.480–0.776)	**<0.001**	0.646 (0.505–0.828)	**0.001**
Laterality				
Right	1			
Left	1.080 (0.848–1.376)	0.532	NA	NA
Bilateral	0.836 (0.309–2.265)	0.724	NA	NA
Histology				
MALT	1			
DLBCL	2.548 (1.852–3.505)	**<0.001**	1.900 (1.3702.636)	**<0.001**
FL	1.572 (1.141–2.165)	**0.006**	1.189 (0.860–1.644)	0.295
Other	1.605 (1.133–2.275)	**0.008**	1.657 (1.157–2.373)	**0.006**
Year of diagnosis				
1998–2003	1		1	
2004–2009	0.815 (0.620–1.073)	0.145	0.976 (0.737–1.294)	0.868
2010–2015	0.531 (0.345–0.817)	**0.004**	0.536 (0.347–0.829)	**0.005**
Methods				
No surgery	1		1	
Local tumor excision	0.813 (0.568–1.163)	0.257	0.947 (0.655–1.37)	0.773
Superficial parotidectomy	0.687 (0.490–0.966)	**0.031**	0.697 (0.492–0.989)	**0.043**
Total parotidectomy	0.598 (0.437–0.818)	**0.001**	0.682 (0.492–0.945)	**0.021**
Radiation				
No	1			
Yes	0.800 (0.629–1.019)	0.070	NA	NA
Chemotherapy				
No	1			
Yes	0.948 (0.730–1.232)	0.691	NA	NA

**Table 5:** Univariate and multivariate analysis of cancer-specific survival.

Characteristics	Univariate analysis HR (95% CI)	*p*	Multivariate analysis HR (95% CI)	*p*
Age				
≤65	1			
>65	3.475 (2.323–5.198)	**<0.001**	3.634 (2.389–5.528)	**<0.001**
Sex				
Female	1			
Male	1.126 (0.772–1.641)	0.538	NA	NA
Race				
White	1			
Black	1.040 (0.557–1.940)	0.902	NA	NA
Other	0.343 (0.109–1.083)	0.068	NA	NA
Marital status				
No married	1			
Married	0.549 (0.378–0.795)	**0.002**	0.663 (0.453–0.971)	**0.035**
Laterality				
Right	1			
Left	1.432 (0.976–2.099)	0.066	NA	NA
Bilateral	1.764 (0.547–5.688)	0.342	NA	NA
Histology				
MALT	1			
DLBCL	4.045 (2.413–6.783)	**<0.001**	3.471 (2.036–5.917)	**<0.001**
FL	1.973 (1.131–3.442)	**0.017**	1.665 (0.951–2.916)	0.074
Other	3.630 (2.166–6.083)	**<0.001**	3.513 (2.061–5.989)	**<0.001**
Year of diagnosis				
1998–2003	1			
2004–2009	0.746 (0.493–1.128)	0.165	0.827 (0.542–1.262)	0.401
2010–2015	0.410 (0.218–0.772)	**0.006**	0.397 (0.209–0.753)	**0.005**
Methods				
No surgery	1			
Local tumor excision	0.727 (0.420–1.258)	0.255	0.935 (0.53–1.647)	0.815
Superficial parotidectomy	0.717 (0.436–1.179)	0.190	0.840 (0.506–1.395)	0.502
Total parotidectomy	0.429 (0.258–0.715)	**0.001**	0.573 (0.339–0.968)	**0.037**
Radiation				
No	1			
Yes	0.663 (0.454–0.969)	**0.034**	0.607 (0.410–0.898)	**0.012**
Chemotherapy				
No	1			
Yes	1.287 (0.876–1.891)	0.198	NA	NA

**Table 6:** Impact of surgical techniques on OS and CSS.

	Characteristics	Overall survival HR (95% CI)	*p*	Cancer-specific survival HR (95% CI)	*p*
Before PSM	No surgery	1		1	
	Local tumor excision	0.813 (0.568–1.163)	0.257	0.727 (0.420–1.258)	0.255
	Superficial parotidectomy	0.699 (0.498–0.982)	**0.039**	0.726 (0.441–1.194)	0.207
	Total parotidectomy	0.592 (0.432–0.809)	**0.001**	0.426 (0.256–0.709)	**0.001**

After PSM	No surgery	1		1	
	Local tumor excision	0.686 (0.400–1.179)	0.173	0.377 (0.134–1.062)	0.065
	Superficial parotidectomy	0.563 (0.318–0.996)	**0.048**	0.444 (0.174–1.133)	0.089
	Total parotidectomy	0.533 (0.319–0.892)	**0.017**	0.319 (0.125–0.815)	**0.017**

## Data Availability

The data supporting the study's findings are freely available in the Surveillance, Epidemiology, and End Results' Program at https://seer.cancer.gov/.
